# Fever of Unknown Origin: The Workup and Diagnosis of Pel-Ebstein Fever

**DOI:** 10.7759/cureus.21959

**Published:** 2022-02-06

**Authors:** Prachi Khanna, Natalie Malluru, Raaj Pyada, Mitul Gupta, Kartik Akkihal, Thomas C Varkey

**Affiliations:** 1 Internal Medicine, Dell Medical School, The University of Texas at Austin, Austin, USA; 2 Kinesiology and Health Education, College of Education, University of Texas at Austin, Austin, USA; 3 Business Management, Grand Canyon University, Phoenix, USA

**Keywords:** differential for fever of unknown origin, lymphoma, fever of unknown origin, hodgkin's lymphoma, pel-ebstein

## Abstract

The clinical and diagnostic workup of fever of unknown origin (FUO) is key in the treatment of patients on the internal medicine service. In this article, the authors present a case of fever of unknown origin, walk through the differential diagnosis, explain the laboratory testing ordered in the workup of the patient as well as the resulting values of said testing, and discuss the pathophysiology and diagnostic criteria for the diagnosis of Pel-Ebstein fever. The authors also discuss a clinical pearl when working with electronic health records to ensure that the needs of the patient in question are met.

## Introduction

Hodgkin’s lymphoma, a cancer of the lymphatic system, is characterized by genetic mutations leading to the proliferation of abnormally oversized lymphocytes [1,2]. Hodgkin’s is distinguished from non-Hodgkin’s by the presence of Reed-Sternberg cells, abnormal multinucleated lymphocytes [1,2]. Pel-Ebstein fever is a rare condition reported in patients with Hodgkin’s lymphoma, characterized by cyclic fevers that rise and fall every one or two weeks. Due to the rarity of disorder, many patients often suffer from lack of treatment or experience difficulties receiving sufficient care for their Pel-Ebstein fevers [1,2]. The authors provided a clinical case study, clinical workup, and discussed treatment for a patient suffering from Pel-Ebstein fevers secondary to Hodgkin’s lymphoma.

## Case presentation

The patient is a 53-year-old woman with a medical history of hypothyroidism, eczema, type II diabetes mellitus, and a reported history of non-Hodgkin’s lymphoma as indicated in the chart on previous hospital admission in March 2020, who presented with chief complaints of fever and a dry non-productive cough. She was sent to the emergency room directly from the infusion center where she was due to receive chemotherapy due to her low white blood cell count and increased temperature found at the clinic. The patient stated that for the last two and a half weeks she had been experiencing cough, intermittent fevers, diagnosed with pneumonia, and received treatment of a combination therapy consisting of 1 g of ceftriaxone twice daily and 750 mg of azithromycin once daily, for seven days at an outside hospital. The patient endorsed clear rhinorrhea but denied dysuria, diarrhea, shortness of breath, productive cough, constipation, or bleeding from any source. She denied any sick contacts stating that she lives alone but added that she does see her sister often. The patient reported that she had not yet received her coronavirus disease 2019 (COVID-19) vaccination due to her chemotherapy schedule. The patient had a past surgical history of cholecystectomy for gallstones, appendectomy for appendicitis, hysterectomy for uterine polyps, and port placement to facilitate chemotherapy infusions. The patient reported drug allergies to lisinopril, penicillin, and sulfa antibiotics which all resulted in whole body pruritus. The patient’s home medication regimen can be found in Table 1.

**Table 1:** Patient’s home medication regimen. Contained within this table are the medications, dosage, dosing interval, and indication for each medication that she was taking at home.

Medication	Dosage and Frequency	Indication
Acyclovir	1200 mg taken twice daily by mouth	Viral illness prophylaxis
Ondansetron	8 mg as needed every eight hours to be taken by mouth	Chemotherapy associated nausea
Potassium chloride	20 mEq taken twice daily by mouth	To replace and maintain her potassium blood levels
Naproxen	500 mg taken twice daily by mouth	For aches and pains
Benzonatate	200 mg taken once daily by mouth	Cough suppressant
Cyclobenzaprine	5 mg by mouth as needed at bedtime	To help induce sleep
Dupixent	300 mg by subcutaneous injection every two weeks	To mitigate eczema symptoms
Gabapentin	300 mg by mouth three times a day	For neuropathic pain symptoms
Glipizide	10 mg by mouth once a day	For diabetes mellitus management
Hydroxyzine	10 mg by mouth at bedtime	To help induce sleep
Levothyroxine	137 mcg by mouth in the morning	To manage her hypothyroid symptoms
Metformin	500 mg in the morning and 1000 mg at dinner by mouth	To manage her diabetes mellitus
Guaifenesin	600 mg by mouth twice a day	To manage mucus secretions

On physical examination, the patient was febrile to 100.7℉, but all other vital signs were within normal limits. She had some small strands of hair that were visible on the top of her head but was otherwise bald. There were no signs of erythema, swelling, induration, lymphadenopathy, or other signs of skin or tissue infection. Apart from her fever, the entirety of her physical examination was within normal limits.

Differential diagnosis

The differential for fever of unknown origin (FUO) includes concurrent infection, malignancy, non-infectious inflammatory disease, as well as other lesser likely conditions such as drug-induced fever or factitious fever [3-9]. Please see Table 2 for an evidence-based differential of FUO. Infections are found to be the most common cause of FUO [3-9]. Bacterial and viral susceptibilities are potentially increased given the patient’s ongoing chemotherapeutic treatment [3-9]. While the patient’s reported history of non-Hodgkin’s lymphoma makes this diagnosis likely, other possible etiologies must be considered and eliminated before continuing immunocompromising treatment. Based on the patient’s medical history, care and attention should be drawn to her levothyroxine, a potential iatrogenic cause, which could lead to increased temperatures. However, without other signs and symptoms of systemic disease, this iatrogenic cause is considered less likely. Based on the patient’s objective data, it was unlikely that FUO was factitious or a secondary reaction to a drug.

**Table 2:** Common causes of fever of unknown origin. This information has been compiled from references [4-13] and is based on the currently available prevalence data.

Subgroup	Cause
Infection (20-40%)	Bacterial
	Abdominal or pelvic abscesses
	Dental abscesses
	Endocarditis
	Sinusitis
	Tuberculosis (especially extrapulmonary/disseminated)
	Urinary tract infection
	Viral
	Cytomegalovirus
	Epstein-Barr virus
Malignancy (20-30%)	Colorectal cancer
	Leukemia
	Lymphoma (Hodgkin and non-Hodgkin)
Non-infectious inflammatory disease (10-30%)	Connective tissue diseases
	Adult Still disease
	Rheumatoid arthritis
	Systemic lupus erythematosus
	Granulomatous disease
	Crohn disease
	Sarcoidosis
	Vasculitis syndromes
	Giant cell arteritis
	Polymyalgia rheumatica/temporal arteritis
Miscellaneous (10-20%)	Drug-induced
	Factitious fever
	Thromboembolic disease
	Thyroiditis

Laboratory testing

Due to the patient’s recent history of chemotherapy treatment for cancer, the internal medicine team ordered several tests to rule out infection as the main cause of her fevers. The first set of tests included complete blood count (CBC) with differential, complete metabolic panel (CMP), chest x-ray, urinalysis with microscopy and culture, and three sets of blood cultures from two separate sites testing for bacterial, mycobacterial, and fungal infection. The bone marrow biopsy procedure was performed by the interventional radiology team. As per the pathologist, the patient’s biopsy was indicative of Hodgkin's lymphoma - a surprise finding that led to an electronic medical record chart review. The pathologist sent her bone marrow for further evaluation of infection and found that there were no infectious materials. All laboratory results and their corresponding normal values can be found in Table 3.

**Table 3:** Pertinent laboratory and imaging results.

Laboratory/Imaging Test	Subtest	Result	Normal Range
Complete blood count	White blood cell count	2.2 thousand/mm^3^	4.5-11.0 thousand/mm^3^
	Hemoglobin	8.6 g/dL	12-14 g/dL
	Differential - lymphocytes	6% of all white blood cells	30-40% of all white blood cells
	Differential - neutrophils	52.6% of all white blood cells	50-65% of all white blood cells
Blood culture	N/A	No growth	No growth
Complete metabolic panel	Sodium	127 mEq/L	135-145 mEq/L
Chest x-ray	N/A	Normal chest anatomy	Normal chest anatomy
Urinalysis	Gross urinalysis	Normal light-yellow coloration	Normal light-yellow coloration
	Dipstick	Negative	Negative
	Microscopy	Negative	Negative
	Urine culture	No growth	No growth
	Spot urine sodium	83 mmol/L	
	Spot urine osmolality	712 mOsm/kg	
Karius Test	N/A	Negative	Negative
Bone marrow biopsy	N/A	Hodgkin’s lymphoma	Normal hematopoietic stem cells

Diagnosis and treatment

Throughout the workup of her fevers, the patient had episodes where her fevers spiked to 104℉ while her systolic blood pressure dropped into 90s and her diastolic blood pressure dropped into 50s. These factors necessitated transfer to the medical intensive care unit (MICU). Nevertheless, within hours the fever broke and her blood pressure normalized. These fever and shock episodes happened cyclically, with episodes happening every two to three days, raising the suspicion of a non-infectious etiology [4-13]. To ensure the patient would not be treated prematurely with immunocompromising drugs, the internal medicine team and the infectious disease team stated that it would be wise to await the results of the Karius Test.

While waiting eight days for the Karius Test results, the team began treatment of the patient’s hyponatremia. The low salt was deemed to be the result of a syndrome of inappropriate antidiuretic hormone (SIADH) caused by her Hodgkin’s lymphoma. She was treated with 5 mg midodrine by mouth three times a day, 1.2 L fluid restriction daily, and 1000 mg sodium chloride tablets twice daily to bring her sodium levels within normal range.

Based on the patient’s medical history, negative laboratory results, and lack of infectious agent, the most likely etiology of her fevers was determined to be Pel-Ebstein fevers. Because of the lack of infection, the oncology team stated that they felt comfortable in moving forward with providing the patient with chemotherapy for her Hodgkin’s lymphoma. They started her on an intravenous infusion regimen of gemcitabine (​​1 g/m^2^) and oxaliplatin (75 mg/m^2^) for her Hodgkin’s lymphoma. This second-line treatment regimen was chosen because the patient had failed earlier treatment with the preferred first-line treatment of adriamycin, bleomycin, vinblastine, and dacarbazine.

The patient remained in the hospital for two additional days, after completing her chemotherapy regimen, to be monitored for unusual reactions to her chemotherapy. She was discharged when her absolute neutrophil count (ANC) was above 500 cells/mm^3^ and she was fever-free for 24 hours. Her total stay, including the workup, fever-shock episodes, chemotherapy, and recovery, was 14 days from initial admission.

## Discussion

FUO is defined as the temperature of 38.3℃ (100.9℉) or higher, which occurs for greater than three weeks with no known cause after investigation [3]. The most common etiologies are infection, malignancies, non-infectious inflammatory disease, and other miscellaneous causes [3]. The diagnostic workup for FUO centers around searching for potential diagnostic clues (PDCs), which can guide the team towards a potential etiology and treatment plan. The initial approach involves taking comprehensive history, physical examination, and appropriate lab testing [3-9]. The preliminary lab investigations should include complete blood count, liver function tests, erythrocyte sedimentation rate, urinalysis, and basic cultures. Specific techniques and imaging should be based on the PDCs, whereas more invasive techniques (i.e., lumbar puncture or bone marrow biopsy) should only be performed when there is a specific need or if an extensive investigation has failed to reveal a diagnosis [3-9].

While the patient’s complete history, physical examination, and laboratory results failed to yield an infectious agent, the bone marrow biopsy indicated that the patient had Hodgkin’s lymphoma. Therefore, the most likely etiology of her fevers was determined to be Pel-Ebstein fever, which is a rare but documented phenomenon associated with Hodgkin’s lymphoma [14-18]. Some estimates state that roughly 35% of those afflicted with Hodgkin’s lymphoma experience this phenomenon [14-18]. In this condition, patients experience cycles of high temperature that typically last one-to-two weeks, followed by afebrile periods of similar duration. It is not unusual for patients with both Hodgkin’s and non-Hodgkin’s lymphoma to present with elevated daily temperatures, as it is theorized that in certain malignancies, such as lymphomas, there is a release of pyrogenic cytokines from tumor cells or macrophages, such as interleukin-1 (IL-1), interleukin-6 (IL-6), and tumor necrosis factor-alpha (TNF-α). These cytokines affect the thermostatic set point by inducing prostaglandin E2, which directly acts on the hypothalamus [17]. However, the exact mechanism behind the cyclic nature of Pel-Ebstein fever is still unknown and the phenomenon itself and its association with Hodgkin’s lymphoma is widely debated [18]. While more data and inquisitorial work must be done on the topic, FUO’s relatively consistent association with Hodgkin’s lymphoma leads the team to believe that Pel-Ebstein fevers are a true phenomenon that can therefore be treated.

One of the key pieces that became increasingly salient to the case was the patient’s misdiagnosis in the chart of “non-Hodgkin’s lymphoma.” When looking through the notes later, it was determined that the initial mistake had been entered into her chart during one of her previous admissions to the hospital. Following the biopsy which demonstrated Hodgkin’s lymphoma and the revelation of the error within the electronic medical record, the record was amended to ensure that the patient would be able to be treated appropriately and that the medical record was now clear as to her specific type of malignant disease. Herein, the authorial team wanted to stress the importance of verifying the information with the patient and with previous records to ensure accuracy. Nevertheless, human errors can (and often do) lead to the entry of “chart lore” [19,20]. Therefore, it is key for clinicians to be on the proverbial “lookout” for these errors and be ready to make amendments to ensure accuracy in the record [19,20].

## Conclusions

This case study discussed the care and management of a patient with a fever of unknown origin, complicated by an error in the patient’s chart history which misreported the patient’s diagnosis and treatment of Hodgkin’s lymphoma as non-Hodgkin’s lymphoma. The patient was managed conservatively and thoroughly tested until the most likely cause of her symptoms was found to be Pel-Ebstein fever associated with Hodgkin’s lymphoma. A key clinical practice takeaway from this case is the importance of verifying the accuracy of patient charts with patients and previous records.
