# Correction: Fever of Unknown Origin: The Workup and Diagnosis of Pel-Ebstein Fever

**DOI:** 10.7759/cureus.c63

**Published:** 2022-03-29

**Authors:** Prachi Khanna, Natalie Malluru, Raaj Pyada, Mitul Gupta, Kartik Akkihal, Thomas C Varkey

**Affiliations:** 1 Internal Medicine, Dell Medical School, The University of Texas at Austin, Austin, USA; 2 Kinesiology and Health Education, College of Education, University of Texas at Austin, Austin, USA; 3 Business Management, Grand Canyon University, Phoenix, USA

The original published version of this article incorrectly referred to the Karius Test as a PCR test. The Karius Test is a non-invasive blood test utilizing metagenomic sequencing of microbial cell-free DNA, not a PCR test. Three instances within the article where the Karius Test was referred to or labeled as a PCR test have been updated as a result. The authors regret that this error was not identified and corrected prior to publication.

